# Association of *CETP*, *APOA5*, *IL6*, and *PON1* Gene Variants with Dyslipidemia and Cardiovascular Risk in a Population from Cauca Department, Colombia

**DOI:** 10.3390/genes16050545

**Published:** 2025-04-30

**Authors:** Astrid Lorena Urbano-Cano, Rosa Elvira Álvarez-Rosero, Yamil Liscano

**Affiliations:** 1 Grupo de Investigación en Salud Integral (GISI), Departamento Facultad de Salud, Universidad Santiago de Cali, Cali 760035, Colombia; yamil.liscano00@usc.edu.co; 2 Grupo de Investigación en Genética Humana Aplicada (GIGHA), Departamento de Ciencias Fisiológicas, Universidad del Cauca, Popayan 190003, Colombia

**Keywords:** genetic polymorphisms, cardiovascular risk, *PON1*, *APOA5*, *IL6*, *CETP*

## Abstract

Background: Cardiovascular disease remains the leading cause of death worldwide, and dyslipidemia is a critical, modifiable risk factor. Aim: We sought to evaluate the relationship between polymorphisms in *CETP* (rs3764261), *APOA5* (rs662799), *IL6* (rs1800796), and *PON1* (Q192R) and lipid parameters, and to assess their contribution to dyslipidemia and overall cardiovascular risk in an urban cohort from Cauca, Colombia. Methods: In this cross-sectional observational study, 304 participants aged 40–69 years were enrolled. Clinical, anthropometric, and biochemical data were collected, and genotyping was performed for the four target polymorphisms. We used descriptive statistics to characterize the sample, non-parametric tests to compare lipid levels by genotype, and multivariable logistic regression to identify independent predictors of dyslipidemia. Results: Individuals with dyslipidemia exhibited significantly higher total cholesterol and VLDL levels, lower HDL levels, and an elevated Castelli II index compared with the non-dyslipidemia group. Although *CETP* genotype frequencies differed between groups, only the *APOA5* rs662799 variant was significantly associated with increased VLDL levels, suggesting its potential role as a genetic biomarker of cardiovascular risk. Conclusions: Our findings underscore the interplay between metabolic factors and genetic variants in the pathogenesis of dyslipidemia. Notably, the *APOA5* rs662799 polymorphism emerged as a key determinant of VLDL concentration, highlighting its promise for personalized cardiovascular risk stratification and management in this population.

## 1. Introduction

Cardiovascular diseases (CVDs) remain the leading global cause of death, accounting for 20.5 million deaths in 2021, with 80% occurring in low- and middle-income countries. Hypertension, dyslipidemia, obesity, and inactivity are major contributors, with hypertension alone responsible for over 10 million deaths annually [1,2]. While statins are widely used, newer treatments such as PCSK9 inhibitors and bempedoic acid are essential for achieving optimal cholesterol control [3]. In Latin America, CVDs represent a significant burden on healthcare systems, underlining the urgent need to investigate genetic factors influencing their development. For instance, studies in Colombia have revealed that 46% of participants suffered from hypertension and 65.9% from abdominal obesity, demonstrating the necessity for targeted interventions [4,5]. Furthermore, central obesity, lifestyle factors like alcohol consumption, and dyslipidemia are widespread across the region [6,7]. In Colombia, over 56.6% of the population reports being physically inactive, while 35.3% exhibits signs of dyslipidemia. These factors affects 52.7% of the population, contributing significantly to the cardiovascular health crisis, and underscoring the need for urgent public health interventions to address these modifiable risk factors [8]. The diverse ethnic composition of Latin America, with varying ancestral backgrounds, may further influence the population’s susceptibility to CVD [9], emphasizing the need for tailored healthcare strategies [2]. It is crucial to explore the genetic foundations of CVD, as genetic factors significantly influence its development.

Numerous genes and polymorphisms are associated with increased cardiovascular risk. For example, polymorphisms in genes such as *PON1* (Q192R), *APOA5* (A/G), *IL6* (G/C), and *CETP* (C/A) have been associated with alterations in lipid metabolism, inflammation, and cardiovascular risk [3,10,11]. Studies, both globally and in Latin America, have shown these polymorphisms’ diverse effects. In Colombian populations, the *PON1* Q192R polymorphism has been linked to both decreased susceptibility to coronary artery disease and increased risk of hypertension, with the distribution of the alleles varying between regions [12,13].

In the context of Colombia, particularly in the Cauca region, the genetic variability among different ethnic groups, including Afro-Colombians, Indigenous peoples, and Mestizos, is significant [12,13]. However, underrepresentation of marginalized racial and ethnic groups in genetic research leads to less accurate polygenic risk scores for these populations [14]. This genetic diversity underscores the importance of studying underrepresented populations, as their genetic makeup may offer unique insights into the mechanisms of CVD [15]. In Cauca, where ethnic diversity is high, investigating polymorphisms like *PON1* Q192R can provide crucial information for personalized cardiovascular risk management.

For example, the *PON1* Q192R polymorphism significantly influences paraoxonase enzyme activity, which protects LDL cholesterol from oxidation, a key factor in atherosclerosis development [16]. Conversely, Iranian studies show that the RR genotype increases the risk of obesity and low HDL-C levels [17]. In Colombian studies, the 192R allele has been linked to reduced coronary artery disease risk, while the 192Q allele has been associated with hypertension [13]. Comparatively, in Peruvian Andean populations, the 192R allele is more prevalent but shows no clear link to lipid profiles [18]. In an Argentinian study, the QQ variant and presence of the M allele in the L55M polymorphism were associated with elevated triglycerides and glucose levels in coronary artery disease patients. Both the Q192R and L55M polymorphisms significantly affected paraoxonase and arylesterase activities, with the RR and LL genotypes showing higher enzyme activities, respectively [19].

These regional studies demonstrate that genetic variations, such as the PON1 Q192R polymorphism, interact with environmental and lifestyle factors, making it essential to investigate population-specific genetic influences on CVDs. In Latin America, further research is needed to explore how these genetic variations, combined with modifiable risk factors like obesity and physical inactivity, contribute to the growing burden of CVDs [12,13]. Mexican studies have revealed that the LM/MM and QQ genotypes of *PON1* polymorphisms are linked to decreased *PON1* activity and obesity risk [20]. Additionally, gene–environment interactions have been observed, with arsenic exposure and the PON1 Q192R polymorphism affecting CVD biomarkers in Mexican women [21].

For instance, the aim of this study is to investigate the association between polymorphisms in *PON1*, *APOA5*, *IL6*, and *CETP* and cardiovascular risk factors in a Colombian population. The results are expected to provide a deeper understanding of the roles that these genes play in cardiovascular risk, and to help identify potential biomarkers for prevention and treatment, contributing to more personalized and effective strategies for managing CVDs.

## 2. Materials and Methods

### 2.1. Study Design and Population

This was a cross-sectional observational study conducted in the department of Cauca, Colombia, aiming to investigate the association between genetic polymorphisms in the *CETP*, *APOA5*, *IL6*, and *PON1* genes and cardiovascular risk factors, such as dyslipidemia, hypertension, and diabetes. The study population consisted of 304 individuals, aged between 40 and 69 years old, for the urban area of Cauca, ensuring a representative sample of the general population in the region.

### 2.2. Inclusion and Exclusion Criteria

Participants were included if they met the following criteria:

- Between 40 and 69 years old.
- Working-age individuals.
- Provided informed consent for genetic, clinical, and paraclinical analysis.

The exclusion criteria were as follows:

- Individuals under 40 years of age.
- Individuals with incomplete data on key variables.
- Pregnant women or individuals with severe chronic illnesses (e.g., cancer, autoimmune diseases) that might confound the analysis.

### 2.3. Data Collection

Demographic and clinical data: All participants were recruited after providing informed consent, and each volunteer was interviewed by a trained health professional to fill out a structured questionnaire to establish socio-demographic characteristics (age, gender, provenance, and occupational status), personal clinical history (dyslipidemia, hypertension, diabetes, body mass index (BMI)), biochemical data (total cholesterol, triglycerides, HDL, VLDL), and smoking habits (never, former, current). All of the questionnaires, procedures, and protocols were reviewed and approved by the ethics committee of the University of Cauca, and the guidelines used in the review were based on the bioethical principles established in the Helsinki Declaration of 1975 and the parameters outlined in Resolution 8430 of the Colombian Ministry of Health in 1993.

Genetic data: Blood samples were collected from all participants, and DNA was extracted using the salting-out method [22]. Genotyping was performed for the following single-nucleotide polymorphisms (SNPs): *PON1* Q192R, *APOA5* (rs662799), *IL6* (rs1800796), and *CETP* (rs3764261). The genomic regions harboring the target polymorphisms were amplified by PCR (BioRad, Popayán City, Colombia) and analyzed using a PCR–RFLP assay) (BioRad, Popayán City, Colombia), according to the manufacturer’s protocol.

### 2.4. Cardiovascular Risk Factor Measurements

Cardiovascular risk factors were assessed by measuring each patient’s height, weight, and resting blood pressure during the examination. Hypertension was defined as a systolic blood pressure ≥ 140 mm Hg and/or a diastolic blood pressure ≥ 90 mm Hg [23].

The calculation of BMI involved dividing weight by squared height (kg/m^2^). The subjects were split into three weight groups: normal weight (BMI < 25), overweight (BMI ≥ 29.99), and obese (BMI ≥ 30). Blood samples were obtained for biochemical analysis, and medical records were examined for clinical diagnosis, in order to confirm the existence of personal risk factors. Therefore, if a subject’s fasting cholesterol was 200 mg/dL or above, their HDL level was 40 mg/dL for men and less than 50 mg/dL for women, or their triglycerides were 150 mg/dL, they were deemed to have dyslipidemia. Dyslipidemia was defined as the presence of one or more abnormal lipid parameters: elevated total cholesterol, low-density lipoprotein cholesterol, or triglycerides, and/or reduced high-density lipoprotein cholesterol, either in isolation or in combination. The cutoff values applied were based on the recommendations established by the Colombian consensus for the diagnosis and management of dyslipidemia in adults, as well as the 2019 ESC/EAS Guidelines for the Management of Dyslipidaemias: Lipid Modification to Reduce Cardiovascular Risk [24,25].

### 2.5. Statistical Analysis and Data Visualization

Data were analyzed using SPSS^®^ version 25 and R version 4.4.1 (both accessed in August 2024). The normality of quantitative variables was initially examined using the Kolmogorov–Smirnov test with a Lilliefors correction. Given that several variables did not meet normality assumptions, quantitative data were expressed as medians with interquartile ranges (IQRs), while categorical variables were summarized as frequencies and percentages. The chi-squared test was used for comparing proportions. For comparisons of continuous variables between two groups, the Mann–Whitney U test was employed, and for comparisons among more than two groups, the Kruskal–Wallis test was applied. A *p*-value < 0.05 was considered to be statistically significant. For the logistic regression models, the overall fit was evaluated using the −2 log-likelihood and the Hosmer–Lemeshow goodness-of-fit test, and explanatory power was assessed using the Cox–Snell R^2^ and Nagelkerke R^2^. Furthermore, the variance inflation factor (VIF) was computed for the variables included in the regression model to assess the presence of multicollinearity among the predictors.

Additionally, the genotype distributions for *IL6_rs1800796*, *PON1_Q192R*, *APOA5_rs662799*, and *CETP* were analyzed to identify differences between individuals with and without dyslipidemia. The allelic and genotypic frequencies were determined by direct counting, and the Hardy–Weinberg equilibrium was evaluated through χ^2^ tests. A separate logistic regression analysis was carried out specifically for *CETP* genotypes to further investigate their association with dyslipidemia. Kruskal–Wallis tests were performed to assess the differences in lipid levels (total cholesterol, HDL, and VLDL) according to each SNP genotype. When significant differences were detected, post hoc comparisons were conducted using the Mann–Whitney U test with Bonferroni correction.

### 2.6. Data Visualization

The results were visualized using R ggplot2 (accessed on August 2024).

## 3. Results

### 3.1. Patient Demographics

A total of 304 individuals were evaluated, divided into a non-dyslipidemia group (n = 116) and those with dyslipidemia (n = 188). The overall median age was 52 years (IQR = 13), with no statistically significant difference between groups. In terms of sex distribution, 53.6% of the participants were female, and no significant difference was observed between the dyslipidemia and non-dyslipidemia groups (see Table 1).

Regarding ethnicity, the majority self-identified as Mestizo (68.8%), followed by White (18.4%) and Afro-descendant (12.8%). A statistically significant difference was noted in the proportion of Mestizos between the two groups: 72.9% of participants with dyslipidemia were Mestizo, compared with 62.1% of the non-dyslipidemia group (*p* = 0.013). Most of the participants were from urban areas (75.7%) and lived with a partner (57.9%), without notable differences between groups.

### 3.2. Clinical Characteristics

BMI was significantly higher in participants with dyslipidemia (median 26.6 vs. 25.4 kg/m^2^, *p* = 0.035). Similarly, participants with dyslipidemia had a significantly lower median systolic blood pressure (135 mmHg) than the non-dyslipidemia group (140 mmHg; *p* = 0.026), while diastolic pressure remained comparable between groups (median 80 mmHg). With respect to biochemical markers, those with dyslipidemia had notably higher total cholesterol and VLDL levels (both *p* < 0.001), as well as lower HDL levels (*p* < 0.001). The percentage of body fat was also significantly greater in the dyslipidemia group (*p* = 0.014) (see Table 1).

Hypertension was highly prevalent in both groups (91.4% overall), showing no significant difference between them (*p* = 0.697). This finding is particularly relevant given the well-established association between hypertension and dyslipidemia. Although no significant differences in the prevalence of hypertension were found between the groups analyzed, the consistently elevated rate across both groups suggests a substantial burden of cardiometabolic risk in this population, which may merit further investigation in future studies.

Likewise, the prevalence of diabetes (26.3% overall) did not differ significantly (*p* = 0.888). Smoking status was similar across groups (*p* = 0.441), and very few participants reported alcohol consumption (2%).

Finally, the group with dyslipidemia included a higher proportion of individuals with a secondary or technical education level (62.2%), whereas the non-dyslipidemia group had more participants with similar educational attainment (75.9%). This difference was statistically significant (*p* = 0.014). Overall, these findings suggest that dyslipidemia is associated with higher BMI, altered lipid profiles, and certain socio-demographic factors, particularly ethnicity and educational level.

### 3.3. Multivariable Logistic Regression Analysis

The multivariable logistic regression model included BMI, systolic blood pressure, cholesterol, HDL, triglycerides, LDL, VLDL, the plasma atherogenic index (AIP), Castelli II indices (for men and women), body fat percentage, ethnicity, and educational level. Among these predictors, cholesterol, HDL, triglycerides, LDL, VLDL, and both Castelli II indices were statistically significant. In particular, cholesterol (OR = 0.966, *p* < 0.001) and VLDL (OR = 0.909, *p* < 0.001) exhibited inverse associations, indicating that lower levels are linked with increased odds of dyslipidemia. Conversely, HDL (OR = 1.077, *p* < 0.001), triglycerides (OR = 7.84, *p* < 0.001), LDL (OR = 3.723, *p* = 0.009), and the Castelli II indices for men (OR = 13.722, *p* < 0.001) and women (OR = 16.786, *p* < 0.001) were positively associated with dyslipidemia. In contrast, BMI, systolic blood pressure, body fat percentage, ethnicity, and educational level did not reach statistical significance (*p* > 0.05) and, thus, do not independently predict dyslipidemia in this model.

In the analysis conducted, the key assumptions underlying multivariable analysis were verified to ensure the validity of the logistic regression model. First, the absence of multicollinearity among the independent variables was confirmed by examining the variance inflation factors, all of which remained below the critical threshold of 5, indicating that the predictors were not excessively correlated, and ensuring the stability and interpretability of the estimated coefficients. The model summary statistics revealed a −2 log-likelihood of 64.664, with a Cox–Snell R^2^ of 0.673 and a Nagelkerke R^2^ of 0.915, reflecting substantial explained variance. The Hosmer–Lemeshow goodness-of-fit test demonstrated excellent model calibration (χ^2^(8) = 1.274, *p* = 0.996). Additionally, the database underwent a continuous quality review during both the data collection and processing phases to ensure data integrity, and to prevent the occurrence of missing values.

These findings underscore the pivotal role of specific lipid parameters and related indices in predicting dyslipidemia within the studied population (see Table 2A).

The integrated multivariable model demonstrated very similar explanatory power to the clinical-only model, with a slight increase in the −2 log-likelihood (66.014 vs. 64.664) and almost identical Cox–Snell and Nagelkerke R^2^ values (0.671 and 0.913 vs. 0.673 and 0.915; see Table 2B). The calibration remained excellent, as evidenced by a non-significant Hosmer–Lemeshow test (χ^2^(8) = 7.286, *p* = 0.506), mirroring the strong fit observed in the clinical-only model.

Among the clinical predictors, total cholesterol, HDL, triglycerides, and VLDL continued to emerge as the strongest independent determinants of dyslipidemia, each retaining statistical significance at *p* < 0.05. Total cholesterol maintained its inverse association (OR = 0.944, 95% CI 0.919, 0.970, *p* < 0.001), while HDL (OR = 0.651, 95% CI 0.472, 0.898, *p* = 0.009), triglycerides (OR = 2.568, 95% CI 1.734, 3.804, *p* < 0.001), and VLDL (OR = 0.878, 95% CI 0.795, 0.971, *p* = 0.011) all remained highly significant. In contrast, LDL lost its significance (OR = 1.015, *p* = 0.643), suggesting that its predictive information largely overlaps with that of other lipid measures. Interestingly, the plasma atherogenic index (AIP) became significant in the integrated model (OR ≈ 0.000, 95% CI 0.000, 0.195, *p* = 0.013), likely due to better adjustment for correlated lipids, whereas both Castelli II indices lost significance when genetic terms were introduced, implying redundancy with the core lipid variables.

None of the four genetic variants (*CETP rs3764261*, *APOA5 rs662799*, *PON1 Q192R*, or *IL6 rs1800796*) reached statistical significance after adjusting for the full set of clinical covariates. Their very wide confidence intervals reflect imprecision, which is most likely attributable to low minor allele frequencies and our sample size. This pattern confirms that while metabolic lipid parameters are the principal drivers of dyslipidemia risk, these particular SNPs do not contribute independent predictive value in this cohort.

Overall, the minimal change in model fit statistics and the excellent calibration of both models indicate that incorporating genetic predictors into the multivariable analysis does not meaningfully enhance performance.

By removing the composite metrics AIP, Castelli II, and LDL to address multicollinearity (VIF  >  5), the refined clinical model (Table 2C) maintained adequate calibration (Hosmer–Lemeshow *p* = 0.281), although it explained slightly less variance (Nagelkerke R^2^ = 0.819 vs. 0.913 in the integrated model, Table 2A). Crucially, the core lipid predictors—HDL (OR 1.122), total cholesterol (OR 0.951), triglycerides (OR 1.522), and VLDL (OR 0.908)—remained highly significant, underscoring the robustness of these basic measures compared to the more complex indices.

This reduction in redundant covariates simplified the model structure and eliminated coefficient instability, yielding reasonable standard errors without diminishing the central role of lipid parameters. Notably, Afro-descendant ethnicity continued to exert a strong, independent effect (OR 8.973, *p* = 0.022), whereas body mass index, body fat percentage, systolic blood pressure, and educational level did not reach statistical significance.

### 3.4. Genetic Associations

#### 3.4.1. Genotypic Distribution

**Figure 1:**
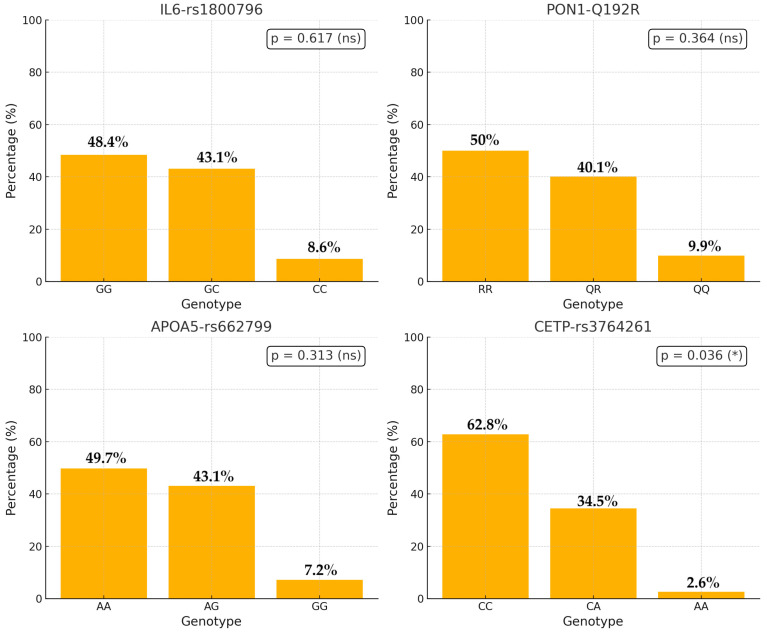
Genotype frequencies for *PON1* Q192R, *APOA5*, *IL6*, and *CETP* gene variants. In all three cases, the differences in genotype distributions between individuals with and without dyslipidemia were not statistically significant (*), suggesting no association with the condition in the studied population.

As depicted in Figure 1, the GG genotype predominates for *IL6 rs 1800796*, with GC as the second most common variant, and CC constituting a minor proportion. A similar distribution can be observed in *PON1*_Q192R, where the RR genotype again exhibits the highest frequency compared to QQ and QR. In APOA5_rs662799, GG remains the most prevalent genotype, followed by lower frequencies of GA and AA. Finally, the *CETP_rs 3764261* distribution also demonstrates CC as the predominant genotype, followed by CA, while AA is the least represented among the study population.

In this comparison of genotype frequencies between individuals with and without dyslipidemia (see Table 3), three of the evaluated polymorphisms (*IL6_1800796*, *PON1_Q192R*, and *APOA5_rs662799*) showed no statistically significant differences (*p* > 0.05). In contrast, the distribution of *CETP* genotypes differed significantly between groups (*p* = 0.036). Specifically, the CC genotype was more prevalent among those without dyslipidemia (71.6% vs. 57.4%), whereas the CA genotype was more common in participants with dyslipidemia (39.4% vs. 26.7%). These findings suggest a possible role of *CETP* genetic variation in susceptibility to dyslipidemia within the studied population.

#### 3.4.2. Association with Lipid Levels

**Figure 2:**
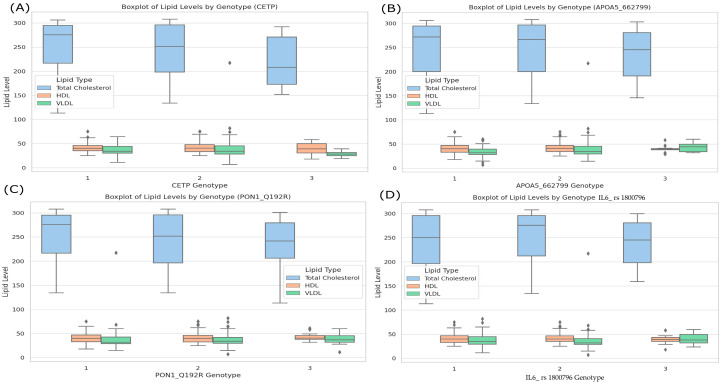
Boxplots of lipid levels (HDL, VLDL, total cholesterol) by genotype. Each panel (**A**–**D**) shows the distribution of total cholesterol, HDL, and VLDL levels for the different genotypes of *CETP_rs 3764261* (**A**), *APOA5_rs 662799* (**B**), *PON1*_Q192R (**C**), and *IL6_rs 1800796* (**D**). In these boxplots, blue corresponds to total cholesterol, orange represents HDL, and green denotes VLDL. The boxes illustrate the medians (horizontal lines) and interquartile ranges, while the whiskers and any points outside them indicate the variability and potential outliers within each genotype group.

In Figure 2, total cholesterol, HDL, and VLDL levels are compared according to the genotypes of *CETP* (A), *APOA5_rs662799* (B), *PON1_Q192R* (C), and *IL6_rs 1800796* (D). Visually, there are no marked differences in the values of total cholesterol, HDL, or VLDL for *CETP*, *PON1*_Q192R, and *IL6_1800796*, as the medians and interquartile ranges largely overlap among the genotypes. However, in the case of *APOA5_rs 662799*, greater variability in VLDL levels can be observed, particularly in one of the genotypes. This finding is in line with the statistical results, which indicate significant differences in this lipid fraction for that polymorphism.

Based on the boxplots in Figure 2, we conducted a sex-stratified χ^2^ analysis of HDL levels across each genotype (Table 4). For *IL6-rs1800796*, male carriers of the GG genotype exhibited a markedly higher prevalence of low HDL compared with females (69.9% vs. 20.3%, *p* < 0.001), a pattern that persisted for both the CC (57.1% vs. 26.8%, *p* < 0.001) and GC genotypes (77.8% vs. 17.6%, *p* = 0.003). Similar significant sex disparities were observed for *PON1*-Q192R: RR homozygotes (58.9% vs. 27.1%, *p* < 0.001), QQ homozygotes (71.1% vs. 17.4%, *p* < 0.001), and QR heterozygotes (66.7% vs. 19.0%, *p* = 0.011) all showed greater low-HDL prevalence in men. Notably, the *APOA5-rs662799* AA and GG genotypes also differed by sex (AA: 59.6% vs. 31.9%, *p* < 0.001; GG: 70.5% vs. 13.2%, *p* < 0.001), as did the AG group (66.7% vs. 6.2%, *p* = 0.003). Finally, the *CETP-rs3764261* genotypes CC, CA, and AA each demonstrated significant male–female differences in HDL status (all *p* < 0.001). We performed identical stratified analyses for cholesterol, triglycerides, LDL, and VLDL levels; however, none demonstrated significant sex–genotype interactions (all *p* > 0.05).

#### 3.4.3. Association Between *CETP* Genotype and Lipid Indices

The analysis in Table 4 examines the association between *CETP* genotype and the Castelli II index (LDL/HDL) in men, using logistic regression. The CC genotype serves as the reference category, with an OR of 1. For the AA genotype, an OR of 1.604 was observed, suggesting a potential 60% increase in the odds of having an elevated Castelli II index compared to the CC group; however, this result was not statistically significant (*p* = 0.528), as indicated by the wide 95% confidence interval (0.370–6.950). Similarly, the CA genotype showed an OR of 1.200, reflecting a 20% increase in the odds relative to the reference group, yet this association was also non-significant (*p* = 0.810), with a broad 95% confidence interval (0.271–5.313). The low Wald statistics for both AA (0.399) and CA (0.058) further support the lack of statistical significance.

Overall, while there appears to be a trend toward higher odds of an abnormal Castelli II index in men with the AA or CA genotypes compared to those with the CC genotype, the results do not provide conclusive evidence of an independent association between *CETP* genotype and the Castelli II index in this population (see Table 5).

The analysis in Table 6 evaluates the association between *CETP* genotype and the Castelli II index (LDL/HDL) in women, using logistic regression. The CC genotype is used as the reference group, with an odds ratio (OR) of 1. For the AA genotype, an OR of 0.399 was found, suggesting that women with the AA genotype may have lower odds of an elevated Castelli II index compared to those with the CC genotype; however, this association was not statistically significant (*p* = 0.752) and was characterized by a wide 95% confidence interval (0.183–3.097). Similarly, the CA genotype exhibited an OR of 0.479, indicating a potential reduction in the odds relative to the reference group, yet this result was also non-significant (*p* = 0.479), with a 95% confidence interval ranging from 0.113 to 2.031. The Wald statistics for both the AA (0.155) and CA (0.998) genotypes further suggest that these variables do not significantly contribute to the model. Both the men’s and women’s models showed almost no explanatory power for the CETP genotype in predicting an elevated Castelli II index. In men, the model’s −2 log-likelihood was 367.908, with R^2^ values of 0.005 (Cox–Snell) and 0.007 (Nagelkerke), and in women it was 404.282 with R^2^ values of 0.012 and 0.016, respectively. The calibration was formally acceptable (Hosmer–Lemeshow *p* = 1.000 in men and perfectly fitted in women), yet neither the AA nor CA genotypes reached significance in either sex (all *p* > 0.5), indicating that *CETP* rs3764261 does not independently influence the LDL/HDL ratio in this cohort.

#### 3.4.4. Association Between *CETP* Genotype and Dyslipidemia

Despite the significant difference in genotype distribution observed in Table 3, the logistic regression analysis (Table 7) indicated no statistically significant associations between the *CETP* AA or CA genotypes and dyslipidemia compared with the CC reference genotype (*p* = 0.314 and *p* = 0.787, respectively). While the AA genotype yielded an OR above 2.0, the wide confidence interval (0.454–11.717) and the *p*-value above 0.05 suggest that this finding is not robust. Similarly, the CA genotype exhibited an OR of 1.257, which also failed to reach statistical significance. These results imply that, although the genotype frequencies of *CETP* differ between individuals with and without dyslipidemia, the adjusted logistic regression does not support an independent effect of *CETP* variants on dyslipidemia risk. This conclusion is supported by the overall evaluation of the logistic regression model: the overall fit of the model, evaluated using the −2 log-likelihood (397.921), is only moderate, and the determination coefficients (Cox–Snell R^2^ = 0.021, Nagelkerke R^2^ = 0.028) indicate that the model explains very little of the variability in dyslipidemia (between 2.1% and 2.8%), which reflects a limited explanatory power. Taken together, the model’s moderate fit and its limited ability to explain variability reinforce the idea that, in the context of this particular model, the *CETP* AA and CA genotypic variables did not emerge as significant independent predictors of dyslipidemia risk.

#### 3.4.5. Evaluation of Lipid Levels by Polymorphism Using Non-Parametric Tests

In Table 8, the results of the Kruskal–Wallis test are presented to evaluate the differences in total cholesterol, HDL, and VLDL levels according to the genotypes of each polymorphism. For the polymorphisms *IL6_1800796*, *PON1*_Q192R, and *CETP*, no significant differences were observed in any of the lipid parameters evaluated (*p* > 0.05 in all comparisons). In contrast, for *APOA5_rs 662799*, a statistically significant difference was found in VLDL levels (*p* = 0.003), while cholesterol (*p* = 0.44) and HDL (*p* = 0.311) levels did not show significant variations among the different genotypes.

Table 9 details the post hoc comparisons using the Mann–Whitney U test for VLDL levels based on the *APOA5_662799* genotypes, applying a Bonferroni correction (α = 0.0167). The results indicate that the difference in VLDL is significant only between the AA and AG genotypes (*p* = 0.001), while the comparisons of AA vs. GG (*p* = 0.07) and AA vs. AG (*p* = 0.029) did not reach statistical significance. These findings suggest that the *APOA5_662799* polymorphism influences the variation in VLDL levels, with a notable difference between the RR and QR genotypes.

## 4. Discussion

### 4.1. Main Findings

This study examined the clinical, biochemical, and genetic determinants of dyslipidemia in 304 urban Colombian adults. Dyslipidemic participants had higher total cholesterol and VLDL, lower HDL, and greater body fat percentages than those without dyslipidemia. In the fully integrated model (Table 2B), the core lipid parameters—total cholesterol (OR 0.944, 95% CI 0.919–0.970), HDL (OR 0.651, 95% CI 0.472–0.898), triglycerides (OR 2.568, 95% CI 1.734–3.804), and VLDL (OR 0.878, 95% CI 0.795–0.971)—remained the only significant predictors, whereas none of the *APOA5*, *CETP*, *PON1*, or *IL6* variants contributed independently (Table 2B). To address multicollinearity (VIF > 5 for AIP, Castelli II, and LDL), we refitted a parsimonious clinical-only model (Table 2C) omitting those composite indices. This refined model retained excellent calibration (Hosmer–Lemeshow *p* = 0.281) and confirmed HDL (OR 1.122, 95% CI 1.063–1.184), total cholesterol (OR 0.951, 95% CI 0.933–0.969), triglycerides (OR 1.522, 95% CI 1.355–1.709), and VLDL (OR 0.908, 95% CI 0.857–0.962) as robust independent predictors, while body mass index, percentage body fat, systolic blood pressure, and educational level were not significant. Afro-descendant ethnicity emerged as an independent risk factor (OR 8.973, 95% CI 1.376–58.495; *p* = 0.022). Taken together, these results underscore the central role of basic lipid parameters in dyslipidemia risk and demonstrate that composite indices and the tested genetic variants add no incremental predictive value in this cohort.

Previous studies have demonstrated that the minor allele frequency of the rs3764261 A allele is higher among individuals with a normal or low body mass index. This single-nucleotide polymorphism is located in the *CETP* gene, which encodes the cholesteryl ester transfer protein—an essential component of lipid metabolism that mediates the transfer of cholesteryl esters from HDL to apolipoprotein B-containing lipoproteins. The rs3764261 A allele was also significantly associated with increased lean body mass (OR = 2.38, 95% CI = 1.30–4.34). Genetic polymorphisms in *CETP* have been linked to interindividual differences in lipid profiles and body composition. These findings suggest that the association with *CETP* gene polymorphisms may be modulated by additional factors, including age, body mass index, and circulating lipid levels [26].

Regarding genetic associations, our findings indicate a significant difference in *CETP* genotype distribution between individuals with and without dyslipidemia, with the AA genotype being more prevalent in the dyslipidemia group. However, logistic regression analysis did not support an independent effect of *CETP* genotypes on dyslipidemia risk. Similarly, while *CETP* genotypes showed some association with the Castelli II index in men and women, these relationships were not statistically significant. The loss of statistical significance for the *CETP* variant in the multivariable analysis, despite the significance observed in univariate comparisons, reflects an example of confounding in statistical modeling. In the univariate analysis, the *CETP* genotype was significantly associated with the outcome; however, this association may have been partially driven by confounding variables simultaneously related to both the *CETP* genotype and the outcome, such as age. If older individuals show a higher frequency of the *CETP* variant and also tend to experience worse outcomes independently of genotype, failing to adjust for age may falsely attribute the effect to the *CETP* variant. After controlling for these confounders in the multivariable model, the true independent association of the *CETP* variant was clarified, resulting in the loss of statistical significance. This finding underscores the critical importance of multivariable adjustment in genetic association studies to avoid biased interpretations.

The only genetic marker that showed a significant impact on lipid levels was the *APOA5_rs 662799* polymorphism, which was associated with increased VLDL levels. Post hoc analysis further confirmed that the RR and QR genotypes exhibited the most pronounced differences in VLDL levels. There have been few studies in adult populations demonstrating that the presence of the C allele of the rs 662799 polymorphism in the *APOA5* gene is associated with dyslipidemia. Several studies have corroborated the association between the *APOA5 (rs662799)* polymorphism and elevated plasma triglyceride levels across diverse populations. Furthermore, this allelic variant of the *APOA5* gene has been linked to an increased susceptibility to dyslipidemia [27].

From these findings, we can highlight the multifactorial nature of dyslipidemia, where both genetic predisposition and metabolic factors interact to determine disease risk. While *CETP* variants may contribute to lipid metabolism, their specific role in dyslipidemia remains uncertain, warranting further investigation in larger, multiethnic cohorts [28,29].

#### Biological Mechanisms of *APOA5*_rs 662799 and Dyslipidemia

A growing body of evidence underscores the critical function of *APOA5* in maintaining lipid homeostasis, particularly in the regulation of plasma triglyceride concentrations. The *APOA5* gene encodes apolipoprotein A-V, which exerts a modulatory effect on both the synthesis and catabolism of triglyceride-rich lipoproteins such as VLDL [30]. APOA5 is a novel apolipoprotein that significantly influences serum triglyceride levels. Mouse model studies show that its overexpression lowers TG levels, while its absence increases them. This highlights APOA5′s essential regulatory role in triglyceride metabolism [31]. Additionally, APOA5 has been shown to be associated with elevated fasting triglyceride levels. These findings suggest a potential triglyceride-independent role for APOA5 in the pathogenesis of cardiovascular disease [32].

Variations in APOA5, exemplified by the APOA5_rs 662799 polymorphism, are believed to alter the gene’s expression levels or the resulting protein’s conformation, ultimately impairing lipoprotein lipase (LPL) activity and compromising the clearance of VLDL particles from the bloodstream [33,34]. Not only does apolipoprotein A-V appear to promote the LPL-mediated hydrolysis of triglycerides, it also influences hepatic VLDL secretion, a process whose disruption may drive increases in circulating triglycerides and VLDL. Over time, such persistently elevated levels of triglyceride-rich lipoproteins can foster an atherogenic lipid profile, thereby escalating the risk of cardiovascular diseases. Although direct functional assays were beyond the scope of this study, the consistent association between APOA5_rs 662799 and elevated VLDL accords with these mechanistic postulates, suggesting that this polymorphism plays a substantial role in dyslipidemia’s pathogenesis [35,36,37].

Nevertheless, further investigation is warranted to fully delineate the molecular underpinnings of *APOA5_rs 662799*. Future endeavors could integrate state-of-the-art proteomics and metabolomics technologies to pinpoint how this polymorphism modifies *APOA5* function and VLDL metabolism. Such studies may clarify whether alterations in enzyme-binding affinity, post-translational processing, or protein stability are key drivers of elevated VLDL levels in carriers of the variant allele [35,38]. Complementing these mechanistic studies, a recent genome-wide association analysis of gene–environment interactions in four Korean cohorts (n = 18,025) confirmed *APOA5* as a lipid-associated locus whose effects on triglyceride levels are modulated by adiposity. This work highlights APOA5′s critical role in the obesity-sensitive regulation of lipid metabolism and its contribution to dyslipidemia’s heritability [39].

### 4.2. Comparison with Other Studies

When comparing these findings with similar studies conducted previously in other regions of Latin America and globally (see Table 10), several relevant considerations emerge.

This study was conducted in the context of previous research, including that of Vinueza et al. (2010) [40] and Ponte-Negretti et al. (2017) [41], which included over 11,000 participants from multiple Latin American cities. The larger sample sizes of these previous studies facilitated broader epidemiological evaluations and allowed for more robust estimations of dyslipidemia’s prevalence. This comparison underscores the methodological importance of large-scale studies for accurately generalizing genetic and metabolic findings across diverse contexts.

The multi-gene approach taken in this study, simultaneously examining *CETP*, *APOA5*, *IL6*, and *PON1*, contrasts with those of earlier studies that typically focused on single polymorphisms. For instance, Giraldo et al. (2012) [42] exclusively studied *CETP* variants, while Carranza Alva et al. (2017) [18] analyzed only the *PON1* Q192R polymorphism. This study’s approach thus provides deeper insights into the complex interactions underpinning dyslipidemia and cardiovascular risk, reflecting the multifactorial nature of these conditions.

Despite identifying significant differences in *CETP* genotype distributions between participants with and without dyslipidemia, this study determined that these variants were not independent predictors after controlling for other clinical and metabolic factors. These findings closely align with those of Giraldo et al. (2012) [42], who also found no independent associations between *CETP* polymorphisms and coronary artery obstruction severity in a comparable Colombian cohort, highlighting the limited predictive capacity of isolated genetic variants without considering broader metabolic contexts.

Environmental and lifestyle factors strongly influence genetic expression, as demonstrated by Siller-López et al. (2017) [13] in Colombian coffee harvesters. Their findings indicated significant gene–environment interactions, where the *PON1* Q192R variant was linked with hypertension and cardiovascular risk, possibly mediated by occupational pesticide exposure. This study similarly emphasizes the necessity of considering lifestyle and environmental modulators when interpreting genetic risks, particularly regarding *CETP* and *APOA5* variants, indicating that genetic predisposition may be significantly influenced by contextual factors.

Interestingly, results regarding the *PON1* Q192R polymorphism vary significantly across studies. Corredor-Orlandelli et al. (2021) [12] reported a protective association of the 192R allele against acute coronary syndrome in Bogotá, a finding that was not clearly replicated in this study. Such discrepancies likely reflect regional and ethnic variations, highlighting the importance of conducting population-specific studies, particularly in regions like Cauca, characterized by significant ethnic diversity and a predominantly indigenous population.

International comparisons further highlight the significance of ancestry in genetic manifestations of cardiovascular diseases. Rai et al. (2021) [43] noted significant associations of the *IL6*-174 G/C polymorphism with cardiovascular disease in predominantly Asian populations. In contrast, this study found no such significant relationship in the Cauca population, reinforcing the necessity for ancestry-specific studies, since global findings may not directly generalize across genetically diverse Latin American populations.

Moreover, the findings of Moreno-Godínez et al. (2019) [20], who studied Mexican adults, indicated significant influences of *PON1* polymorphisms on enzymatic activities associated with cardiovascular risk. This study observed partial consistency but found no significant association of *PON1* variants with specific lipid parameters such as HDL and VLDL, suggesting that population-specific or environmental modulation effects might be influential.

Further emphasizing genetic diversity within Colombian populations, Puerto-Baracaldo et al. (2024) [44] investigated adults with severe hypertriglyceridemia across multiple Colombian cities. Their cross-sectional genetic sequencing study identified 92 genetic variants, including 18 novel variants primarily associated with the LMF1 gene. Notably, genetic variations in LMF1 were significantly correlated with severe hypertriglyceridemia, especially among individuals with pancreatitis, who exhibited markedly elevated triglyceride levels. These results align with this study’s findings, underscoring the importance of investigating multiple genetic factors simultaneously to comprehensively understand the pathogenesis of dyslipidemia within Colombia.

The integrative approach adopted in this study, combining clinical, biochemical, and genetic data, aligns with the recent trend exemplified by Rodríguez-Gutiérrez et al. (2023) [45] in Mexico. Such comprehensive assessments are critical for understanding the multifaceted nature of dyslipidemia. Collectively, these studies suggest that genetic factors alone have modest predictive power, highlighting the necessity of incorporating lifestyle and metabolic factors into strategies for effective prevention and treatment in diverse Latin American populations.

**Table 10:** Comparative studies on genetic variants and cardiovascular risk in Latin American and global populations (2010–2025).

Study (Author, Year)	Population	Location	Sample Size (n)	Genes/Polymorphisms Investigated	Study Design/Type	Age (Mean/Range)	Key Outcomes
Rios et al. (2010) [46]	Patients undergoing coronary angiography (African- and Caucasian-Brazilians)	Brazil	667 (253 African; 414 Caucasian)	*IL-1B* (-511C > T) and *IL-6* (-174G/C)	Case–control study	Not specified	In African-Brazilians, IL-1B-511CC and IL-6-174GG genotypes independently predicted CAD risk.
Vinueza et al. (2010) [40]	General adult population assessed for dyslipidemia (CARMELA study)	Multiple Latin American cities (e.g., Barquisimeto, Bogotá, Buenos Aires, Lima, Mexico City, Quito, Santiago)	11,550 (aggregated across cities)	Lipid profile assessment (no specific genes)	Cross-sectional, population-based	25–64 years (adults)	High prevalence of dyslipidemia with low HDL-C and high triglycerides; significant between-city variability.
Giraldo et al. (2012) [42]	Patients with coronary artery disease	Quindío, Colombia	559	*CETP* polymorphisms (TaqIB, MspI, RsaI) and CETP activity	Case–control study	Not specified	No significant association between CETP activity/polymorphisms and the degree of coronary obstruction.
Suárez-Sánchez et al. (2016) [47]	School-aged children (5–14 years)	Mexico (urban settings, recreational facilities)	1559	*APOA5* (rs662799) and *APOA1* (rs5072)	Cross-sectional study (with meta-analysis component)	5–14 years	Significant association between APOA5/APOA1 variants and elevated triglyceride levels, indicating genetic influence on lipid levels.
Carranza Alva et al. (2017) [18]	Clinically healthy adults	Junín district, Peru	79	*PON1* Q192R	Descriptive, cross-sectional	Not specified (adults)	Genotype distribution: QQ 13.9%, QR 45.6%, RR 40.5; high frequency of 192R allele; no association with lipid profile or APOA1 levels.
Siller-López et al. (2017) [13]	Coffee harvesters (occupational group potentially exposed to pesticides)	Central Colombia (Coffee Belt)	205	*PON1* Q192R (rs662)	Cross-sectional occupational study	Not specified	PON1 Q192R genotype frequencies: QQ 38%, QR 44%, RR 18%; association of the 192Q allele with increased hypertension risk and overall cardiovascular risk.
Ponte-Negretti et al. (2017) [41]	General adult population (data aggregated from multiple surveys)	Latin America (various cities)	Data from ~11,550 participants (CARMELA)	Atherogenic dyslipidemia profile (lipid fractions)	Consensus review and epidemiological analysis	25–64 years (adults)	Reported high prevalence of atherogenic dyslipidemia (low HDL-C and high triglycerides) across Latin American cities; provided recommendations for diagnosis/treatment.
Moreno-Godínez et al. (2019) [20]	Adults with cardiovascular risk factors	Various regions, Mexico	~480	*PON1* (L55M and Q192R polymorphisms)	Cross-sectional study	Not specified	Demonstrated associations between PON1 variants and paraoxonase activity; significant correlations with cardiovascular risk factors.
Corredor-Orlandelli et al. (2021) [12]	Patients with acute coronary syndrome (ACS)	Central Colombia (e.g., Bogotá)	163 patients (cases) with controls from gnomAD (n = 17,711)	*PON1* Q192R (rs662)	Retrospective case–control study	Not specified (adults)	Found that the 192R allele was associated with lower CAD risk under a dominant model (OR 0.58; 95% CI 0.42–0.8; *p* < 0.01); no association with platelet reactivity under clopidogrel treatment.
Rai et al. (2021) [43]	Diverse global populations (meta-analysis; focus on ancestral subgroups)	Global (various ancestral groups)	51,213 (CAD endpoint); 6807 (IL-6 levels endpoint)	*IL6*-174 G/C (rs1800795)	Systematic review and meta-analysis	Not applicable	Overall significant association between IL6-174 G/C and CAD, mainly driven by Asian/Asian-Indian groups; “C” allele carriers had higher IL-6 levels.
Rodríguez-Gutiérrez et al. (2023) [45]	Biochemical, clinical, and genetic characteristics of Mexican patients with primary hypertriglyceridemia, including the first case of hyperchylomicronemia syndrome due to GPIHBP1 deficiency	Jalisco, Mexico	58	*APOA5*, GPIHBP1, LMF1, LPL	Cross-sectional study with genetic sequencing	Mean age ~37.5 years (variable among groups)	Detailed biochemical–clinical–genetic characterization of primary hypertriglyceridemia; identified 74 variants; first Mexican case of hyperchylomicronemia syndrome due to GPIHBP1 deficiency.
Puerto-Baracaldo et al. (2024) [44]	Adults with severe hypertriglyceridemia (sHTG)	Multiple regions in Colombia (Bogotá, Bucaramanga, Medellín, Manizales)	166 participants (62% male)	*LPL*, *APOC2*, *APOA5*, *GPIHBP1*, *LMF1*	Cross-sectional genetic sequencing study	Mean age: 50.0 ± 14.1 years	Identified 92 genetic variants; 18 were novel. Pathogenic and likely pathogenic variants found primarily in LMF1. Genetic variation in LMF1 significantly associated with sHTG. Highest ever triglyceride levels, significantly higher among participants with a history of pancreatitis (4317 mg/dL) compared to those without pancreatitis (1769 mg/dL). Suggests that genetic variants, particularly in LMF1, play an important role in the pathogenesis of sHTG in Colombian populations.
This study (2025)	Adults with and without dyslipidemia	Cauca, Colombia	304	*CETP*, *APOA5*, *IL6*, *PON1*	Cross-sectional observational study with multivariable logistic regression	Median age 52 years (IQR = 13)	Found dyslipidemia to be associated with higher BMI and altered lipid profiles; significant differences in CETP genotype distribution between groups, although CETP variants did not independently predict dyslipidemia risk.

### 4.3. Clinical Implications

The findings of this study have significant clinical implications for the early identification and comprehensive management of dyslipidemia. The analysis revealed that specific lipid parameters, such as total cholesterol, HDL, triglycerides, LDL, VLDL, and Castelli II indices, are key predictors of this condition. These results underline the importance of routine and detailed lipid profile monitoring in at-risk populations, particularly in clinical settings with a high prevalence of dyslipidemia. Notably, indices like Castelli II, demonstrating a strong association with dyslipidemia, could significantly enhance clinical practice by enabling early and accurate cardiovascular risk stratification [35,48,49,50].

The identification of significant differences in anthropometric measures such as BMI and body fat percentage, along with demographic variables (ethnicity and educational level), although they were not independent predictors in the multivariable analysis, highlights the importance of comprehensive preventive strategies. These should include not only targeted pharmacological interventions to correct the identified lipid abnormalities but also sustainable lifestyle modifications. Promoting healthy dietary habits, regular physical activity, and targeted health education in specific groups could potentially reduce the incidence and associated complications of dyslipidemia [51,52].

From a genetic perspective, although the *CETP* polymorphism demonstrated significant genotype distribution differences between the dyslipidemia and non-dyslipidemia groups, its independent influence in the adjusted model was not statistically significant. However, the *APOA5_rs 662799* polymorphism showed a robust association with VLDL levels, highlighting its potential clinical relevance as a biomarker in this population. This finding supports integrating targeted genetic assessments to enhance the personalized clinical management of dyslipidemia, paving the way towards precision medicine tailored to individual genetic and metabolic susceptibilities [38,53].

Consequently, the optimal clinical management of dyslipidemia should combine selective genetic evaluations with lifestyle-focused measures, including dietary modifications, weight management, and increased physical activity, alongside appropriate pharmacological interventions when indicated [38]. This integrated, personalized approach may significantly improve cardiovascular health outcomes.

#### Public Health and Preventive Perspective

The present findings have notable ramifications for public health policies focused on preventing cardiovascular diseases in populations exhibiting similar socio-demographic characteristics. The strong association of lipid parameters (total cholesterol, HDL, triglycerides, LDL, VLDL, and Castelli II indices) with dyslipidemia suggests that identifying at-risk groups through routine lipid profiling and targeted screening programs could substantially reduce cardiovascular morbidity and mortality. Early detection, particularly in communities with a high prevalence of dyslipidemia or unique genetic backgrounds, may enable proactive interventions, such as lifestyle counseling, dietary modifications, and precise pharmacological treatments. Additionally, tailoring preventive strategies to the specific needs of various demographic subgroups, especially those differing in ethnicity or educational attainment, could facilitate more equitable health outcomes. By incorporating these data-driven risk assessment tools into existing public health frameworks, clinicians and policymakers can design more efficient screening campaigns, improve health education initiatives, and ultimately mitigate the long-term burden of cardiovascular disease at the population level [54,55,56].

### 4.4. Limitations and Strengths

This study has several methodological limitations that should be considered when interpreting the results. First, the retrospective design, based on medical records, might have introduced biases in the measurement and uniformity of certain variables, potentially limiting the accuracy of key biochemical and lifestyle data. The absence of potentially relevant variables such as D-dimer, which has demonstrated predictive value in other studies, may weaken the analysis’s robustness and reduce the predictive capacity of the model used. Furthermore, the combined analysis of variables collected at admission and during hospitalization could have introduced an immortality bias, potentially distorting the evaluation of temporal and causal associations [57,58,59].

Additionally, it must be acknowledged that the sample size and participant selection may have influenced the generalizability and external validity of the study. Given that the analysis was conducted within a single regional cohort, caution must be exercised when extrapolating these results to broader populations. The lack of external validation further limits the applicability of the findings beyond the studied group. This evidences the critical need for replication in independent cohorts with greater demographic and geographic diversity. Future research should not only focus on validating these associations through larger, multicenter studies but also explore potential regional and ethnic variations in lipid–gene interactions. Such efforts would strengthen the robustness and external applicability of the current findings, ultimately advancing a more comprehensive understanding of the genetic determinants of lipid metabolism across diverse populations.

Nevertheless, this study has significant strengths that warrant acknowledgment. Its comprehensive approach, simultaneously analyzing clinical, anthropometric, biochemical, and genetic variables, provides a more complete and multifaceted understanding of dyslipidemia. This approach allows for the examination of complex interactions between genetic and environmental or metabolic factors, offering a more accurate and realistic perspective on disease determinants.

Another notable strength is the rigorous application of advanced statistical methods, including multivariable logistic regression analyses and non-parametric tests, ensuring methodological validity and result robustness. Additionally, this study provides valuable insights into a population characterized by significant ethnic diversity, helping to address knowledge gaps regarding specific genetic and clinical factors in ethnically diverse regions.

Ultimately, these results underscore the complex etiology of dyslipidemia and highlight the necessity of simultaneously considering multiple genetic and environmental factors to enhance preventive and therapeutic strategies tailored to specific populations.

## 5. Conclusions

This study confirms that both traditional lipid parameters and genetic variants jointly influence dyslipidemia’s development. Total cholesterol, HDL, VLDL, and the Castelli II index proved to be robust predictors, while the *APOA5 rs662799* polymorphism stood out as being significantly associated with elevated VLDL levels, reinforcing its potential as a biomarker for individualized cardiovascular risk assessment. Furthermore, our results highlight the necessity of integrating genetic screening with clinical and metabolic evaluations to optimize prevention and treatment strategies. Although variations in *CETP* genotype distribution were observed between dyslipidemic and non-dyslipidemic individuals, these did not independently predict dyslipidemia after adjusting for other factors, underscoring the complexity of gene–environment interactions. Future research should explore additional genetic markers and modulatory environmental factors to refine personalized approaches, particularly within ethnically diverse populations.

## Figures and Tables

**Table 1:** Demographic and clinical characteristics of the study population.

Variable	Non-Dyslipidemia Group (n = 116)	With Dyslipidemia (n = 188)	Total (n = 304)	*p*-Value	Statistic
Age (Years) (Median ± IQR)	51.0 ± 12	53 ± 14	52 ± 13	0.858	U Mann–Whitney
BMI (kg/m^2^) (Median ± IQR)	25.4 ± 5.4	26.6 ± 6.2	26.1 ± 6.1	0.035	U Mann–Whitney
Systolic Pressure (Median ± IQR)	140 ± 40	135 ± 30	140 ± 30	0.026	U Mann–Whitney
Diastolic Pressure (Median ± IQR)	80 ± 10	80 ± 10	80 ± 10	0.132	U Mann–Whitney
Glycemia (Median ± IQR)	99.0 ± 21.5	99.0 ± 25	99.0 ± 23.0	0.402	U Mann–Whitney
Cholesterol (Median ± IQR)	186 ± 27	256.5 ± 96	200 ± 95.0	<0.001	U Mann–Whitney
HDL (Median ± IQR)	49 ± 11	40 ± 14	43 ± 12.0	<0.001	U Mann–Whitney
VLDL (Median ± IQR)	28 ± 4.0	33.5 ± 14.8	30.0 ± 9.0	<0.001	U Mann–Whitney
Body Fat Percentage (Median ± IQR)	27.0 ± 8.3	28.8 ± 7.3	28.3 ± 7.8	0.014	U Mann–Whitney
Sex (Female) (n, %)	61 (54.3%)	102 (52.6%)	163 (53.6%)	0.777	Chi-squared
Sex (Male) (n, %)	55 (47.4%)	86 (45.7%)	141 (46.4%)		
Ethnicity (n, %)	Mestizo: 72 (62.1%)	Mestizo: 131 (72.9%)	209 (68.8%)	0.013	Chi-squared
	White: 31 (26.7%)	White: 25 (13.3%)	56 (18.4%)		
	Afro-descendant: 13 (11.2%)	Afro-descendant: 26 (13.8%)	39 (12.8%)		
Origin (n, %)	Urban: 86 (74.1%)	Urban: 144 (76.6%)	230 (75.7%)	0.628	Chi-squared
	Rural: 30 (25.9%)	Rural: 44 (23.4%)	74 (24.3%)		
In a Relationship (n, %)	Yes 71 (61.2%)	Yes 105 (55.9%)	176 (57.9%)	0.358	Chi-squared
	No: 45 (38.8%)	No: 83 (44.1%)	128 (42.1%)		
Educational Level (n, %)	Secondary and technical education: 88 (75.9%)	Secondary and technical education: 117 (62.2%)	205 (67.4%)	0.014	Chi-squared
	Professional: 28 (24.1%)	Professional: 71 (37.8%)	99 (32.6%)		
Hypertension (n, %)	Yes 107 (92.2%)	Yes 171 (91%)	278 (91.4%)	0.697	Chi-squared
	No: 9 (7.8%)	No: 17 (9%)	26 (8.6%)		
Diabetes (n, %)	Yes 30 (25.9%)	Yes 50 (26.6%)	80 (26.3%)	0.888	Chi-squared
	No: 86 (74.1%)	No: 138 (73.4%)	224 (73.7%)		
Smokers (n, %)	Yes 70 (60.3%)	Yes 105 (55.9%)	175 (57.6%)	0.441	Chi-squared
	No: 46 (39.7%)	No: 83 (44.1%)	129 (42.4%)		
Drinkers (n, %)	Yes 1 (0.9%)	Yes 5 (2.7%)	6 (2%)	0.274	Chi-squared
	No: 115 (99.1%)	No: 183 (97.3%)	298 (98%)		

**Table 2:** (**A**) Multivariable logistic regression model of factors associated with dyslipidemia. (**B**) Integrated multivariable model with clinical and genetic predictors. (**C**) Parsimonious multivariable logistic regression model of clinical predictors.

(A)					
Variables	Standard Error	OR	Wald	95% CI (Lower–Upper)	*p*-Value
BMI (kg/m^2^)	0.069	1.064	0.808	0.929–1.219	0.369
Systolic Pressure	0.008	1.011	1.985	0.996–1.026	0.159
Cholesterol	0.005	0.966	42.927	0.956–0.976	<0.001
HDL	0.021	1.077	12.540	1.033–1.121	<0.001
Triglycerides	0.443	7.84	21.57	3.289–18.708	<0.001
LDL	0.502	3.723	6.850	1.391–9.963	0.009
VLDL	0.025	0.909	15.007	0.866–0.954	<0.001
Plasma Atherogenic Index (AIP) (TG/HDLc)	1.61	1.865	0.288	0.192–18.143	0.591
Castelli II Index = LDL/HDL Men	0.632	13.722	17.160	3.974–47.378	<0.001
Castelli II Index = LDL/HDL Women	0.404	16.786	48.718	7.603–37060	<0.001
Body Fat Percentage	0.048	0.980	0.180	0.893–1.076	0.671
Ethnicity	0.644	3.237	0.073	0.917–11.428	0.068
Educational Level	0.385	1.422	0.835	0.668–3.024	0.361
(**B**)					
**Predictor**	**OR**		**95% CI**		***p*-Value**
Total Cholesterol	0.944		0.919–0.970		<0.001
HDL	0.651		0.472–0.898		0.009
Triglycerides	2.568		1.734–3.804		<0.001
LDL	1.015		0.954–1.079		0.643
VLDL	0.878		0.795–0.971		0.011
AIP	~ 0.000		0.000–0.195		0.013
Castelli II Men	0.282		0.021–3.745		0.337
Castelli II Women	0.066		0.002–2.351		0.136
*CETP rs3764261* (CA vs. CC)	125.695		0.191–82,713.866		0.144
*CETP rs3764261* (AA vs. CC)	19.733		0.027–14,512.118		0.376
*APOA5 rs662799* (AG vs. AA)	68.776		0.137–34,569.115		0.182
*APOA5 rs662799* (GG vs. AA)	187.290		0.371–94,447.467		0.099
*PON1 Q192R* (QR vs. RR)	5.158		0.123–216,509		0.390
*PON1 Q192R* (QQ vs. RR)	3.039		0.060–153,669		0.579
*IL6 rs1800796* (GC vs. GG)	0.023		0.000–14,703		0.251
*IL6 rs1800796* (CC vs. GG)	0.064		0.000–50,292		0.420
(**C**)					
**Predictor**	**OR**		**95% CI**		***p*-Value**
Percentage Body Fat	0.973		0.851–1.112		0.683
Body Mass Index (kg/m^2^)	1.131		0.931–1.374		0.215
VLDL (mg/dL)	0.908		0.857–0.962		0.001
HDL (mg/dL)	1.122		1.063–1.184		<0.001
Total Cholesterol (mg/dL)	0.951		0.933–0.969		<0.001
Ethnicity (Mestizo vs. Reference)	1.796		0.395–8.176		0.449
Ethnicity (Afro-Descendant vs. Reference)	8.973		1.376–58.495		0.022
Educational Level (Sec./Tech. vs. Prof.)	1.245		0.433–3.582		0.684
Systolic Blood Pressure (mm Hg)	1.018		0.997–1.041		0.099
Triglycerides (mg/dL)	1.522		1.355–1.709		<0.001

Note: −2 Log-likelihood: 64.664; Cox–Snell R^2^: 0.673; Nagelkerke R^2^: 0.915; Hosmer–Lemeshow χ^2^(8): 1.274; *p*: 0.996; SE: standard error; OR: odds ratio; Wald: Wald χ^2^ statistic; 95% CI: 95% confidence interval; −2 log-likelihood; 64.664; 66.014; Cox–Snell R^2^/Nagelkerke R^2^; 0.673/0.915; 0.671/0.913; Hosmer–Lemeshow χ^2^ (df)/*p*: 1.274 (8)/0.996; 7.286 (8)/0.506. Model summary: −2 log-likelihood = 123.886; Cox–Snell R^2^ = 0.602; Nagelkerke R^2^ = 0.819; Hosmer–Lemeshow goodness-of-fit: χ^2^(8) = 9.772; *p* = 0.281.

**Table 3:** Distribution of *IL6_rs 1800796*, *PON1_Q192R*, *APOA5_rs 662799*, and *CETP_rs 3764261* genotypes in patients with and without dyslipidemia.

Variable	Non-Dyslipidemia (n = 116)	With Dyslipidemia (n = 188)	Total (n = 304)	*p*-Value	Statistic
*IL6_rs 1800796* (n, %)	GG: 52 (44.8%)	GG: 95 (50.5%)	147 (48.4%)	0.617	Chi-squared
	GC: 53 (45.7%)	GC: 78 (41.5%)	131 (43.1%)		
	CC: 11 (9.5%)	CC: 15 (8.0%)	26 (8.6%)		
*PON1*_Q192R (n, %)	RR: 64 (55.2%)	RR: 88 (46.8%)	152 (50%)	0.364	Chi-squared
	QR: 42 (36.2%)	QR: 80 (42.6%)	122 (40.1%)		
	QQ: 10 (8.6%)	QQ: 20 (10.6%)	30 (9.9%)		
*APOA5_rs 662799* (n, %)	AA: 64 (55.2%)	AA: 87 (46.3%)	151 (49.7%)	0.313	Chi-squared
	AG: 45 (38.8%)	AG: 86 (45.7%)	131 (43.1%)		
	GG 7 (6.0%)	GG: 15 (8.0%)	22 (7.2%)		
*CETP* (n, %)	CC: 83 (71.6%)	CC: 108 (57.4%)	191 (62.8%)	0.036	Chi-squared
	CA: 31 (26.7%)	CA: 74 (39.4%)	105 (34.5%)		
	AA: 2 (1.7%)	AA: 6 (3.2%)	8 (2.6%)		

**Table 4:** Distribution of HDL levels (low vs. high) by genotype and sex.

SNP/Genotype	HDL Level	Male n (%)	Female n (%)	*p*-Value
*IL6-rs1800796 (GG)*	Low	58 (69.9)	13 (20.3)	<0.001
	High	25 (30.1)	51 (79.7)	
*IL6-rs1800796 (CC)*	Low	28 (57.1)	22 (26.8)	<0.001
	High	21 (42.9)	60 (73.2)	
*IL6-rs1800796 (GC)*	Low	7 (77.8)	3 (17.6)	0.003
	High	2 (22.2)	14 (82.4)	
*PON1-Q192R (RR)*	Low	33 (58.9)	26 (27.1)	<0.001
	High	23 (41.1)	70 (72.9)	
*PON1-Q192R (QQ)*	Low	54 (71.1)	8 (17.4)	<0.001
	High	22 (28.9)	38 (82.6)	
*PON1-Q192R (QR)*	Low	6 (66.7)	4 (19.0)	0.011
	High	3 (33.3)	17 (81.0)	
*APOA5-rs662799 (AA)*	Low	34 (59.6)	30 (31.9)	<0.001
	High	23 (40.4)	64 (68.1)	
*APOA5-rs662799 (GG)*	Low	55 (70.5)	7 (13.2)	<0.001
	High	23 (29.5)	46 (86.8)	
*APOA5-rs662799 (AG)*	Low	4 (66.7)	1 (6.2)	0.003
	High	2 (33.3)	15 (93.8)	
*CETP-rs3764261 (CC)*	Low	50 (63.3)	28 (25.0)	<0.001
	High	29 (36.7)	84 (75.0)	
*CETP-rs3764261 (CA)*	Low	38 (70.4)	10 (19.6)	<0.001
	High	16 (29.6)	41 (80.4)	
*CETP-rs3764261 (AA)*	Low	5 (62.5)	–	<0.001
	High	3 (37.5)	–	

SNP: single-nucleotide polymorphism; HDL: high-density lipoprotein; n: number of subjects; %: percentage; *p*: *p*-value.

**Table 5:** Association between *CETP* genotype and Castelli II index (LDL/HDL) in men: Logistic regression results.

*CETP* Genotype	Standard Error	OR	Wald	95% CI (Lower–Upper)	*p*-Value
CC (Reference)	-	1	-	-	-
AA	0.748	1.604	0.399	0.370–6.950	0.528
CA	0.759	1.200	0.058	0.271–5.313	0.810

Model summary: −2 log-likelihood = 367.908; Cox–Snell R^2^ = 0.005; Nagelkerke R^2^ = 0.007. Hosmer–Lemeshow goodness-of-fit: χ^2^(1) = 0.000, *p* = 1.000.

**Table 6:** Association between CETP genotype and Castelli II index (LDL/HDL) in women: Logistic regression results.

*CETP* Genotype	Standard Error	OR	Wald	95% CI (Lower–Upper)	*p*-Value
CC (Reference)	-	1	-	-	-
AA	0.722	0.399	0.155	0.183–3.097	0.752
CA	0.737	0.479	0.998	0.113–2.031	0.479

Model summary: −2 log-likelihood = 404.282; Cox–Snell R^2^ = 0.012; Nagelkerke R^2^ = 0.016. Hosmer–Lemeshow goodness-of-fit: χ^2^(0) = 0.000.

**Table 7:** Association between CETP genotype and dyslipidemia: Logistic regression results.

CETP Genotype	Standard Error	OR	Wald	95% CI (Lower–Upper)	*p*-Value
CC (Reference)	-	1	-	-	-
AA	0.829	2.306	1.014	0.454–11.717	0.314
CA	0.844	1.257	0.073	0.240–6.572	0.787

Model summary: −2 log-likelihood = 397.921; Cox–Snell R^2^ = 0.021; Nagelkerke R^2^ = 0.028. Hosmer–Lemeshow goodness-of-fit: χ^2^(0) = 0.000.

**Table 8:** Kruskal–Wallis test statistics for lipid levels by polymorphism.

Polymorphism	Variable	Kruskal–Wallis H	gl	*p*-Value
*IL6_rs 1800796* (n, %)	Cholesterol	0.005	2	0.997
	HDL	0.804	2	0.669
	VLDL	1.017	2	0.601
*PON1_Q192R* (n, %)	Cholesterol	0.129	2	0.937
	HDL	0.259	2	0.879
	VLDL	2.992	2	0.224
*APOA5_rs662799* (n, %)	Cholesterol	1.641	2	0.44
	HDL	2.336	2	0.311
	VLDL	11.675	2	0.003
*CETP_rs3764261* (n, %)	Cholesterol	1.141	2	0.565
	HDL	0.153	2	0.927
	VLDL	3.472	2	0.176

**Table 9:** Post hoc comparisons (Mann–Whitney U) of VLDL levels by *APOA5_rs662799* genotypes (Bonferroni α = 0.0167).

Comparison	*p*-Value (Mann–Whitney U)	Significant (α = 0.0167)
AA vs. GG	0.07	No
AA vs. AG	0.001	Yes
GG vs. AG	0.029	No

## Data Availability

Data are contained within the article.
